# Identification of Novel LCN2 Inhibitors Based on Construction of Pharmacophore Models and Screening of Marine Compound Libraries by Fragment Design

**DOI:** 10.3390/md23010024

**Published:** 2025-01-05

**Authors:** Ningying Zheng, Xuan Li, Nan Zhou, Lianxiang Luo

**Affiliations:** The Marine Biomedical Research Institute of Guangdong Zhanjiang, School of Ocean and Tropical Medicine, Guangdong Medical University, Zhanjiang 524023, China; a1256912489@gdmu.edu.cn (N.Z.); x.li@gdmu.cn (X.L.); zaonan@gdmu.edu.cn (N.Z.)

**Keywords:** marine natural compounds, LCN2, pharmacophore modeling, scaffold hopping, virtual screening, molecular dynamics simulation

## Abstract

LCN2, a member of the lipocalin family, is associated with various tumors and inflammatory conditions. Despite the availability of known inhibitors, none have been approved for clinical use. In this study, marine compounds were screened for their ability to inhibit LCN2 using pharmacophore models. Six compounds were optimized for protein binding after being docked against the positive control Compound A. Two compounds showed promising results in ADMET screening. Molecular dynamics simulations were utilized to predict binding mechanisms, with Compound 69081_50 identified as a potential LCN2 inhibitor. MM-PBSA analysis revealed key amino acid residues that are involved in interactions, suggesting that Compound 69081_50 could be a candidate for drug development.

## 1. Introduction

Lipocalin-2, also known as siderocalin or neutrophil gelatinase-associated lipocalin (NGAL), is a flexible soluble protein that is involved in various biological functions. It helps in the movement of hydrophobic molecules across cell membranes, regulates the immune response to bacterial infections, promotes the differentiation of epithelial cells, and regulates iron levels. This complex protein was first discovered in the specific granules of human neutrophils [1,2]. LCN2, part of the renowned lipocalin superfamily, is named for its striking similarity in structure to other members of the lipocalin family. This superfamily is known for its tertiary structural features that are similar to lipid transport proteins, consisting of eight reverse-parallel β-sheets organized to create a cup-shaped cavity that acts as a binding site for small lipophilic molecules [3]. LCN2 is expressed in various cells such as hepatocytes, activated lymphocytes, different epithelial cells, and osteoblasts. However, it is believed that white adipose tissue is the main source of LCN2.

LCN2 is involved in various important physiological and pathological processes. It helps to regulate cellular iron levels, can either promote or inhibit apoptosis, and influences the immune response. Research has shown that LCN2 is expressed abnormally in diseases such as endometrial cancer, liver fibrosis, epilepsy, and pulmonary hypertension, suggesting a potential role in their development [4,5,6,7,8,9,10,11,12,13]. Studies have shown that LCN2 has the ability to stimulate vascular endothelial growth factor production, promote angiogenesis, induce EMT, and facilitate cell migration and invasion [4]. Additionally, LCN2 is implicated in numerous signaling pathways, including PI3K/AKT/NF-κB, HIF-1α/Erk, and MafG/MYH9-LCN2 [4,10,14]. Notably, the silencing of LCN2 has been shown to ameliorate inflammatory activation [8]. The development of therapeutic agents targeting LCN2 is likely to be clinically significant for treating various diseases. While several LCN2-related chemicals have been identified, the lengthy drug development process and complex procedures for chemical extraction and synthesis have made it challenging to find inhibitor molecules with clear clinical benefits. Therefore, it is important to invest more resources in discovering efficient and stable inhibitors that can aid in drug screening against LCN2 targets.

In recent years, there has been a noticeable increase in scientific research dedicated to marine life forms such as algae, sponges, corals, and sea squirts. This surge is driven by the desire to uncover the diverse array of natural products found in these organisms. The focus has shifted towards exploring the unique chemical structures, impressive biological activities, and promising medicinal properties of marine secondary metabolites [15]; numerous investigations have confirmed that compounds sourced from marine organisms possess remarkable antitumor, antithrombotic, antibacterial, and anti-inflammatory properties, exhibiting potent bioactivities [16]. This has sparked the potential to explore and screen for inhibitors of LCN2 activity, featuring innovative structural scaffolds [17,18,19]. In order to enhance the scope of our marine compound sourcing, we have combined three databases dedicated to marine natural products, thereby expanding our pool of resources. Specifically, we have integrated the Marine Natural Products Database (MNPD), the Comprehensive Marine Natural Products Database (CMNPD), and the Seaweed Metabolism Database (SWMD) for this study, each contributing to the strength of our data compilation. Our goal is to utilize the vast resources of the ocean to conduct a screening process for new LCN2 inhibitors.

The aim of this study was to discover new and powerful inhibitors of LCN2. To achieve this goal, we first gathered a list of LCN2 inhibitors and then evaluated and selected two pharmacophore models based on receptor–ligand complexes using Discovery Studio 2019. These models were used to screen and assess candidates from a large marine combinatorial library. Following this, a structure-based virtual screening was carried out using two different molecular docking programs, Schrödinger Maestro 11.8 and Discovery Studio 2019. The top 40 compounds with the highest docking scores in both programs were selected for further analysis. Fragment substitution was performed on six small molecules, and the resulting compounds were compared through molecular docking. Two compounds, Compound 69081_38 and Compound 69081_50, were found to have favorable ADMET properties. Molecular dynamics simulations and MM-PBSA calculations were then conducted on these two candidate compounds to assess their interactions and stability. Ultimately, it was determined that Compound 69081_50 shows promise as an effective LCN2 inhibitor.

## 2. Results

### 2.1. Establishment and Validation of Pharmacophore Modeling

Pharmacophore refers to the “pharmacophore elements” and their spatial arrangement in the active molecule of a drug, which play an important role in the activity. Pharmacophore elements maintains the structural characteristics required for the activity of the compound [20]. There are seven main types of pharmacophore elements, including hydrogen bond donors, hydrogen bond acceptors, positive and negative charge centers, aromatic ring centers, hydrophobic groups, hydrophilic groups, and geometric conformational volume impulses. In order to verify the reliability of the constructed pharmacophore model, the ROC curve was applied to verify that the pharmacophore model of the two established in this study has a good ability to differentiate between active and inactive molecules before performing the database screening. The subject operating characteristic curve (ROC curve for short) is also known as the sensitivity curve. The ROC curve graph is a curve that reflects the relationship between sensitivity and specificity. The X-axis of the horizontal coordinate is the 1-specificity, also known as the false-positive rate (specificity), and, the closer the X-axis is to zero, the higher the accuracy; the Y-axis of the vertical coordinate is known as the sensitivity, also known as the true-positive rate (sensitivity), and, the larger the Y-axis is, the higher the accuracy. The entire graph is divided into two parts based on the position of the curve. The area under the curve is called the AUC (area under the curve) and is used to indicate the accuracy of the prediction. The higher the AUC value, the larger the area under the curve, indicating that, the higher the prediction result, the higher the accuracy. The closer the curve is to the upper left corner (the smaller the X, the larger the Y), the higher the accuracy of the prediction.

**Figure 1:**
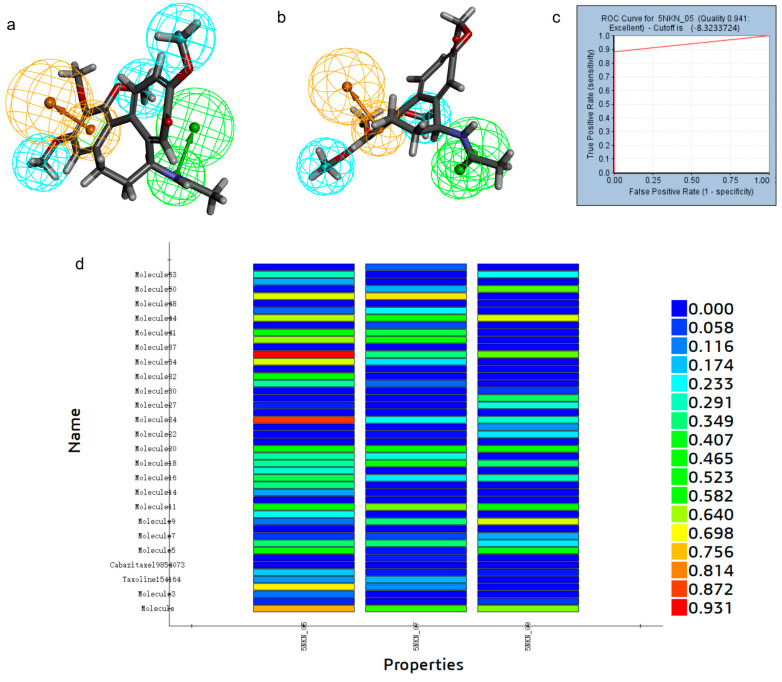
Hydrophobic group features are represented by blue spheres, aromatic ring features are represented by orange spheres, and hydrogen bond donor features are represented by green spheres. (**a**) RL_5, RL_7, and RL_9 pharmacophores. (**b**) Pharmacophore of RL_5. (**c**) ROC curve of RL_5. (**d**) Comparative fitness plots of active small molecules of RL_5, RL_7, and RL_9.

**Figure 2:**
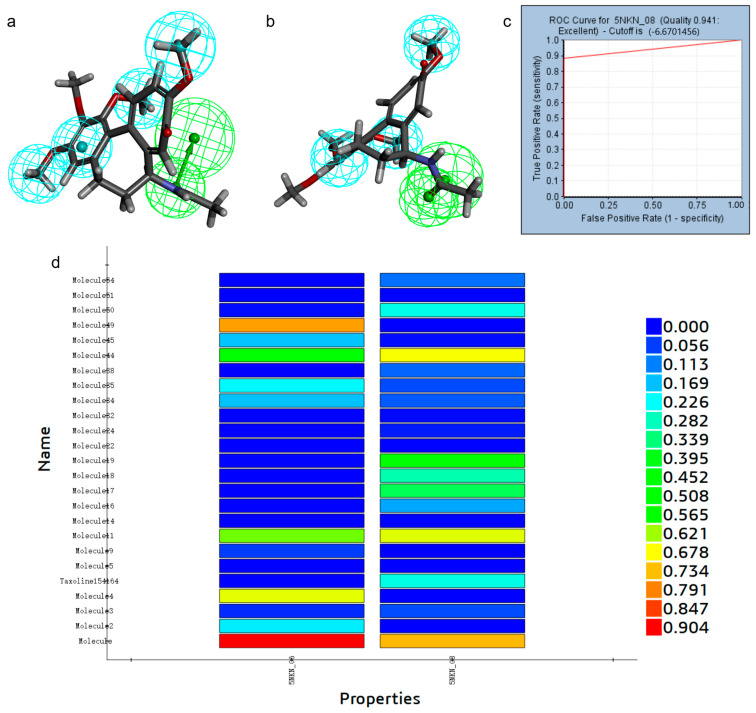
Hydrophobic group features are indicated by blue spheres and hydrogen bond donor features are indicated by green spheres. (**a**) RL_6, RL_8, and RL_10 pharmacophores. (**b**) RL_8 pharmacophore. (**c**) ROC curve of RL_8. (**d**) Comparative fitness plots of active small molecules of RL_6, RL_8, and RL_10.

Using the Discovery Studio platform, we constructed 10 pharmacophore models based on the acceptor-ligand complexes based on the 3D structure of LCN2 protein (PDBID: 5NKN) and its proto-ligand. After the validation of the pharmacophore models, among these 10 pharmacophore models, 6 had better results, namely, RL_5, RL_6, RL_7, RL_8, RL_9, and RL_10, as shown in Figure 1. The synthesized validation results, as shown in Table 1, selected the pharmacophore with sensitivity, specificity, and ROC curve scores all greater than 0.8 and better results in Feature Set and Sensitivity Score; the above six pharmacophores were qualified. RL_5, RL_7, and RL_9’s Feature Sets were all AHHR, and the Feature Sets of RL_6, RL_8, and RL_10 were all AHHH; we selected one pharmacophore from each of the above two groups for virtual screening. We put the 17 active small molecules collected previously into the pharmacophore for fitness comparison, and using Ligand Profiler in Discovery Studio. In the group with Feature Set of AHHR, RL_5 has the highest fitness, as in Figure 1d; in the group with Feature Set of AHHH, RL_8 has the highest fitness, as in Figure 2d. Therefore, we selected RL_5 and RL_8 as the pharmacophores for virtual screening.

### 2.2. Pharmacophore-Based Virtual Screening

RL_5 and RL_8 were imported into Discovery Studio, and 52,765 marine natural compounds were previously pre-processed, utilizing the Ligand Pharmacophore Mapping module in Discovery Studio 2019. First, the marine natural compound library was screened using RL_5, resulting in 9349 remaining small molecules. Then, RL_8 was utilized to screen the remaining molecules, and the final 4922 small molecules were used for the next step.

### 2.3. Docking

Molecular docking is the ideal technique for investigating the optimal binding mode of a compound to a target. Therefore, in order to further screen compounds with good target inhibitory activity, we used the Ligand Docking module of Schrödinger Maestro 11.8 and the Libdock module of Discovery Studio to screen 4922 small molecules with promising potentials for drug discovery against LCN2 protein (PDB ID: 5NKN).

The Schrödinger docking results demonstrated that 4883 small molecules could be successfully docked, of which 230 compounds exhibited superior docking results compared to the positive control Compound A (LibDockScore = −10.994). Based on the principle that lower Schrödinger docking result scores corresponded to better activity, we selected the top 40 compounds with the best docking scores, among which the best score was −14.382 and the lowest score was −12.319.

The Discovery Studio docking results demonstrated that 4922 small molecules could be successfully docked, of which 1230 compounds exhibited superior docking results compared to the positive control Compound A (LibDockScore = 108.36). According to the principle that the higher score for Discovery Studio docking results corresponds to better activity, we selected the top 40 compounds with the best docking scores, among which the best score was 171.933 and the lowest score was 146.222.

By screening the top 40 compounds with the best docking scores from the 2 software programs, it was found that only 6 compounds could pass the docking analysis of the 2 different docking programs at the same time, as shown in Table 2, namely, Compound 50616, Compound 50617, Compound 50618, Compound 44879, Compound 46563, and Compound 69081. Thus, these six compounds have great potential to be LCN2 inhibitors.

### 2.4. Fragment Optimization

Skeleton jumping involves the substitution of the central skeleton of a ligand with a new moiety that possesses comparable functionalities, aimed at improving the properties of an existing compound or discovering entirely unprecedented compounds exhibiting similar functions. By applying this method, based on a full consideration of activity, it is possible to design completely new drug molecules that break through patent protection, have novel structures, and may improve pharmacokinetic properties.

**Figure 3:**
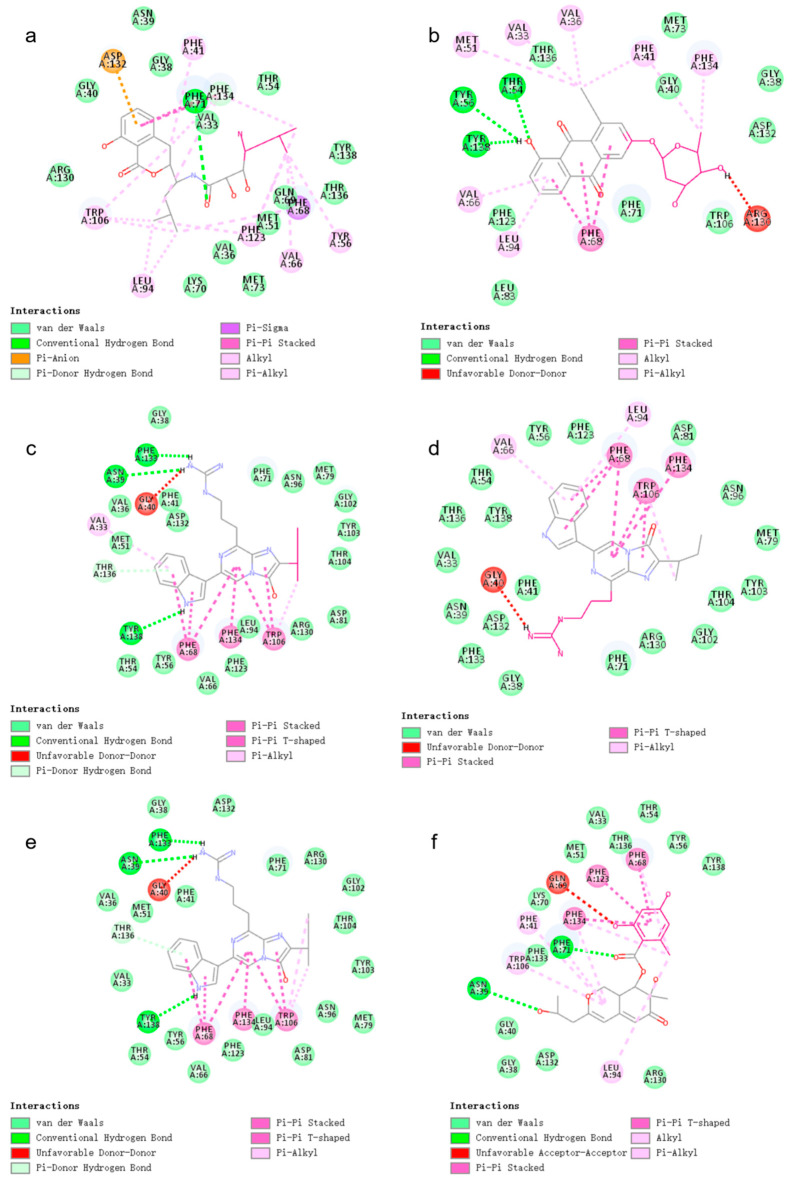
(**a**) 2D interaction diagram of Compound 44879 with LCN2. The rosy-red part is 2-methylpropan-1-amine. (**b**) 2D interaction diagram of Compound 46563 with LCN2. The rosy-red portion is 3,6-dimethoxy-2-methyltetrahydro-2H-pyran-4-ol. (**c**) 2D interaction plot of Compound 50616 with LCN2. The rosy-red part is isopentane. (**d**) 2D interaction diagram of Compound 50617 with LCN2. (Z)-2-methyl-1-propylguanidine is the rosy-red portion. (**e**) 2D interaction diagram of Compound 50618 with LCN2. The rosy-red portion is 1,1-dimethyl-3-propylguanidine. (**f**) 2D interaction diagram of Compound 69081 with LCN2. The rosy-red portion is 3-methoxy-5-methylphenol.

According to the analysis of docking results, 2-methylpropan-1-amine (2-methylpropan-1-amine) in Compound 44879 did not form hydrogen bonding with the receptor and the fragment was small, so we replaced this fragment, as shown in Figure 3a. After the replacement, a total of three new small molecules were generated.

According to the analysis of docking results, 3,6-dimethoxy-2-methyltetrahydro-2H-pyran-4-ol (3,6-dimethoxy-2-methyltetrahydro-2H-pyran-4-ol) in Compound 46563 formed an unfavorable collision with ARG130, so we replaced this fragment, as shown in Figure 3b. After the replacement, a total of 18 new small molecules were generated.

According to the analysis of docking results, 1,1-dimethyl-3-propylguanidine (1,1-dimethyl-3-propylguanidine) in Compound 50616 formed an unfavorable collision with GLY40, but it also formed hydrogen bonding interactions with PHE133 and ASN39, so we did not select this fragment for replacement. Among them, isopentane (isopentane) forms fewer interactions with the receptor and has no hydrogen bonding interactions, so we selected isopentane (isopentane) for replacement, as shown in Figure 3c. After the replacement, a total of 96 new small molecules were generated.

According to the analysis of docking results, (Z)-2-methyl-1-propylguanidine ((Z)-2-methyl-1-propylguanidine) in Compound 50617 formed an unfavorable collision with GLY40, so we selected this fragment for replacement, as shown in Figure 3d. After the replacement, a total of 98 new small molecules were generated.

According to the analysis of docking results, 1,1-dimethyl-3-propylguanidine in Compound 50618 formed one unfavorable collision with GLY40, so we selected this fragment for replacement, as shown in Figure 3e. After the replacement, a total of 98 new small molecules were generated.

According to the analysis of docking results, 3-methoxy-5-methylphenol (3-methoxy-5-methylphenol) in Compound 69081 formed one unfavorable collision with GLN69, so we selected this fragment for replacement, as shown in Figure 3f. After the replacement, a total of 97 new small molecules were generated.

In summary, the backbone leaping of the six candidate compounds, with the desire to find compounds with better interactions with protein LCN2, yielded 420 results. These compounds were visually inspected and all had better interaction with protein LCN2; compounds labeled as HIT were retrieved and stored for further evaluation.

### 2.5. Docking

Molecular docking tools play a pivotal role in structure-based screening and the examination of interaction forces between protein and ligands. In order to further verify the reliability of our chosen structures, we used Schrödinger 11.8 and Discovery Studio 2019 software for docking, docked 420 small molecules obtained after backbone jump to LCN2, and a total of 420 docking results were obtained. The compounds with better docking scores than those before skeleton jump were screened out, and there were three results, namely, Compound 44879_4, Compound 69081_38, and Compound 69081_50; the skeleton jump subplots of these three compounds are shown in Table 3 and Table 4. By analyzing the two-dimensional interaction diagrams of these five complexes, it can be found that these five compounds are able to form a certain number of hydrogen bonds as well as hydrophobic interactions, etc., with the residues of the LCN2 protein. It is obvious that the abundant types of interactions between these three compounds and proteins make the docking effect better than that of the original compounds. The docking results are deemed reliable based on the interaction analysis, and the selected compounds can thus be subjected to further analysis. This will provide a theoretical basis for the drug development of LCN2.

### 2.6. Absorption, Distribution, Metabolism, Excretion, and Toxicity (ADMET) Analysis

**Figure 4:**
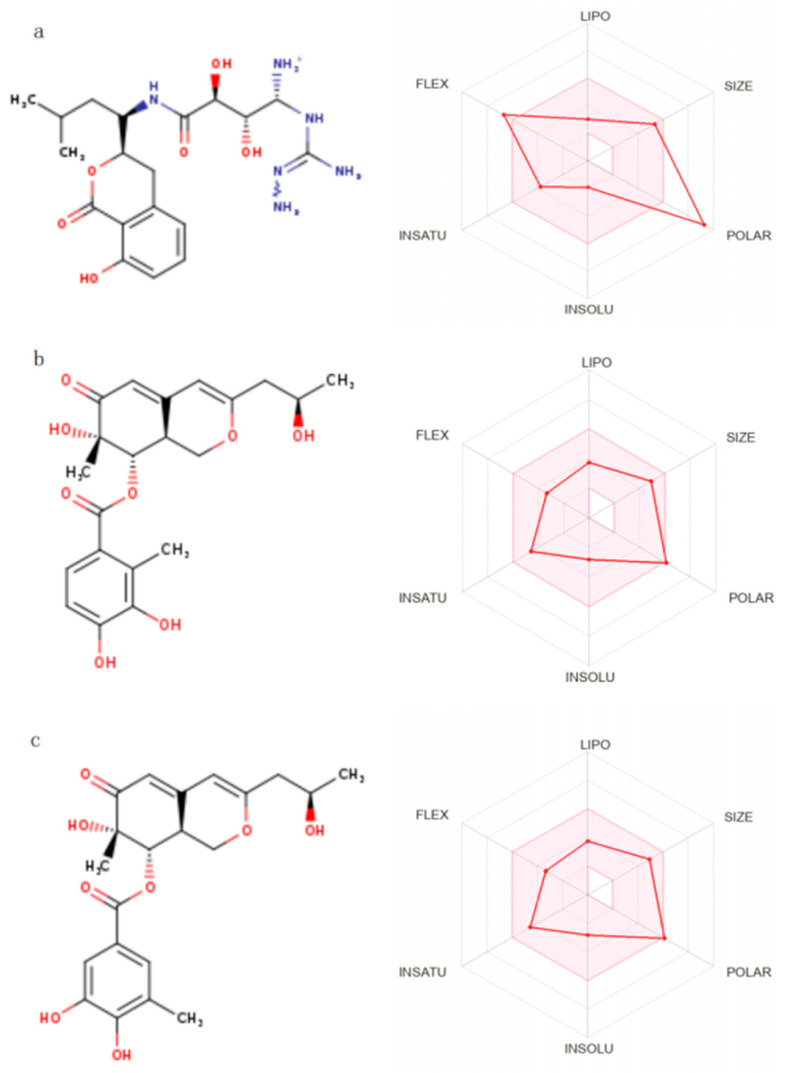
Hexagonal range distribution of ADME properties of candidate Compounds 44879_4, 69081_38, and 69081_50. (**a**) Distribution of ADME properties of Compound 44879_4; (**b**) Distribution of ADME properties of Compound 69081_38; (**c**) Distribution of ADME properties of Compound 69081_50.

Predictions of the biological activity and toxicity of the five compounds from the initial screening were performed using the online tool SwissADME, as shown in Table 5. The table shows only some of the properties of the five compounds obtained from the screening. Of the five compounds selected, all compounds were non-blood–brain barrier penetrating. The Lipinski rule for the evaluation of durability consists of 5 main principles that should be met if a compound is considered to have good formulation properties: relative molecular mass less than 500, number of hydrogen bond donors less than 5, number of hydrogen bond acceptors less than 10, lipid–water partition coefficient less than 5, and number of rotatable bonds less than or equal to 10 [21]. Among the five compounds selected for ADME property analysis, only Compound 69081_38 and Compound 69081_50 met the above five rules and had good gastrointestinal absorption, as shown in Table 5. Compound 69081_38 has a relative molecular mass of 404.41, 4 hydrogen bond donors, 8 hydrogen bond acceptors, 5 rotatable bonds, 5 iLOBs, and 5 rotatable bonds. Compound 69081_38 has a relative molecular mass of 404.41, 4 hydrogen bond donors, 8 hydrogen bond acceptors, 5 rotatable bonds in the compound, an iLOGP value of 2.16, and an ESOL value of −2.83. Compound 69081_50 has a relative molecular mass of 404.41, 4 hydrogen bond donors, 8 hydrogen bond acceptors, 5 rotatable bonds in the compound, an iLOGP value of 2.20, and an ESOL value of −2.83. The two compounds above showed good ADME properties, and, therefore, the ADME screened Compound 69081_50 was found to be good for ADME. The above two compounds showed good ADME properties, so ADME screening resulted in Compound 69081_38 and Compound 69081_50 having good drug-forming properties, as shown in Figure 4, which can be used for the next step of analysis.

### 2.7. Molecular Dynamics

**Figure 5:**
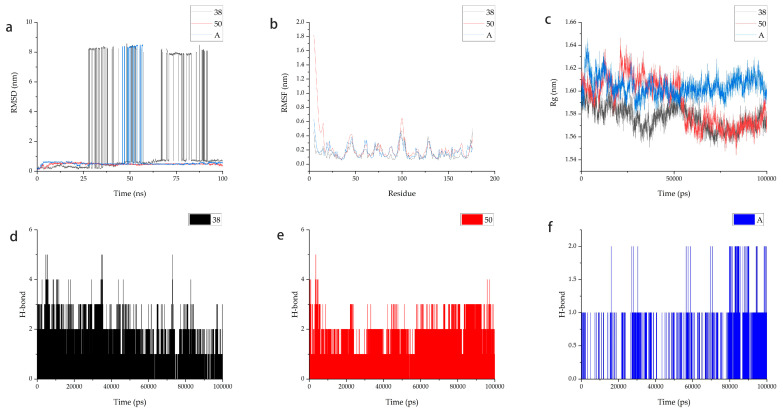
Results of the molecular dynamics simulations of protein–ligand complexes. (**a**) RMSD diagram of protein–ligand complex. (**b**) RMSF diagram of protein–ligand complex. (**c**) Radius of gyration (Rg) graph for complexes with respect to 100 ns of molecular dynamics. (**d**) The hydrogen bond of protein with Compound 69081_38. (**e**) The hydrogen bond of protein with Compound 69081_50. (**f**) The hydrogen bond of protein with Compound A.

RMSD quantifies the deviation of atoms from their mean positions and the extent of their displacement. The root-mean-square deviation (RMSD) of ligands relative to the protein is illustrated in Figure 5a. The RMSD of 69081_50 began to stabilize after 10 ns. The average RMSD from 10 ns to 100 ns was 0.48689. The RMSD of 69081_38 and the RMSD of positive control Compound A are less stable. They all show large fluctuations in 100 ns molecular dynamics simulations. The RMSF of protein residues quantifies the average displacement of these residues from their mean positions within the protein conformation, providing insights into their degree of mobility and thus reflecting the atomic degrees of freedom. As shown in Figure 5b, the RMSF values for complexes ranged from 0.0547 nm to 1.8203 nm. Overall, the RMSF trends were generally consistent, but Compound 69081_50 had higher RMSF values than Compound 69081_38 and positive control Compound A. The RMSD and RMSF data showed an excellent stability of Compound 69081_50 to the protein.

The radius of gyration (Rg) parameter indicates the rigidity and compactness of the complex structures. The g_gyrate tool was used to assess protein compactness for two inhibitors. As depicted in Figure 5c, the protein–ligand complex Rg variation of the three is similar, confined in the range of 1.54413 nm to 1.64644 nm.

Hydrogen bonds are crucial for the stability of protein–ligand complexes and represent the strongest non-covalent interactions. A 100 ns molecular dynamics simulation revealed that LCN2-69081_38 and LCN2-69081_50 displayed a greater count of hydrogen bonds when compared to LCN2-A, in a comparative analysis of hydrogen bond formation in protein–ligand and protein–positive compound complexes. As shown in Figure 5d–f, the average number of hydrogen bonds for LCN2-69081_38 was 1.43071 and 1.42372 for LCN2-69081_50. LCN2-A has the lowest number of hydrogen bonds, with an average of 0.20816. Based on the above analysis, we chose 69081_50 for further research.

### 2.8. MM-PBSA

To authenticate the binding affinity predicted by docking and molecular dynamics simulations, an assessment of the complex’s binding free energy was undertaken. The Poisson–Boltzmann Surface Area (MM-PBSA) methodology serves as an efficacious and trustworthy approach for delving into the intricacies of molecular mechanics, enabling precise computations of the binding free energy in complex systems. The lower the free energy resulting from protein and compound binding, the better the ligand binds to the protein. Based on the results obtained in Table 6, we can observe that all the ligands have negative values. Ligand 69081_50 has a binding energy of −102.785 kJ/mol, which results in a better value compared to A of −76.511 kJ/mol. This is largely influenced by the van der Waals force. Compound 69081_50 is embedded in the pocket of the target active residue, forming more interaction forces with the receptor, further enhancing the binding with the target body. Specifically, the target–ligand interactions are mainly hydrogen-bonding interactions. PHE41, PHE68, PHE71, MET73, TRP106, PHE123, and PHE134 are all critical H-bond residues.

**Figure 6:**
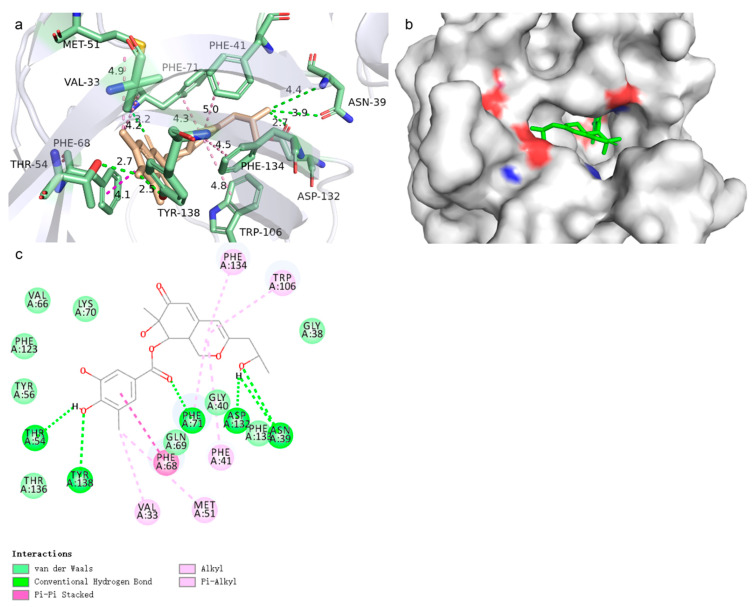
(**a**) Three-dimensional binding pattern of Compound 69081_50 to LCN2 protein. Carbon–hydrogen bonds are shown by pale green dashed lines, hydrogen bonds are shown by green dashed lines, alkyl bonds are shown by pink dashed lines, and Pi interactions are shown by magenta dashed lines. (**b**) The small green molecule is Compound 69081_50, showing the binding of Compound 69081_50 to the LCN2 protein pocket. (**c**) Schematic of the two-dimensional interaction of Compound 69081_50 with the LCN2 protein.

Compound 69081_50, as shown in Figure 6, is anchored at residues VAL33, GLY38-PHE41, MET51, THR54, TYR56, VAL66, PHE68-PHE71, TRP106, PHE123, ASP132-PHE134, THR136, and TYR138, which form in the target active pocket. As can be observed from the three-dimensional structure of the complexes, Compound 69081_50 is substantially embedded in the pocket of the target active residues, which leads to the formation of more van der Waals interactions with the receptor, further enhancing binding to the target. In particular, the interactions between the ligand and the target were predominantly hydrogen bonding interactions, Pi–Pi Stacked, and Alkyl.

It has been mentioned in the literature that LCN2 has a highly similar eight-stranded antiparallel symmetric β-barrel folded structure to lipocalin-type PGD2 synthase protein (L-PGDS), a member of the lipofuscin family, human α1-microglobulin (A1M), and retinol-binding protein 4 (RBP4) [22]. To prevent the potential inhibitor Compound 69081_50 from possibly binding to these compounds and thus causing off-target effects, we docked Compound 69081_50 with the other proteins mentioned above. First, the protein structures of L-PGDS (PDBID: 4ORS), A1M (PDBID: 3QKG), and RBP4 (PDBID: 2WQ9) were downloaded from the PDB website (https://www.rcsb.org/, accessed on 14 December 2024). Then, Discovery Studio was applied to LibDock the Compound 69081_50 to the above three proteins. As shown in Table 7, the docking scores of the potential inhibitor 69081_50 with LCN2 were significantly higher than those of other lipofuscin family members, which indicated the specificity of Compound 69081_50 as an inhibitor of LCN2.

## 3. Discussion

LCN2 is a human lipocalcin 2, also referred to as neutrophil gelatinase-associated lipocalcin. Numerous biological processes have been shown to involve it, encompassing iron and fatty acid transport, cellular migration and survival, inflammatory reactions, and the triggering of apoptosis. LCN2 is also a key factor in various signaling pathways such as PI3K/AKT/NF-κB, HIF-1α/Erk. and MafG/MYH9-LCN2, which create conditions for tumorigenesis and progression [4,10]. This has prompted further research into LCN2 inhibitors. In 2020, LCN2 inhibitors designed with doxorubicin as a scaffold based on a molecular docking approach were reported. These inhibitors have inhibited the progression of LCN2-associated diseases [23]. Despite the existence of numerous LCN2-inhibitory compounds that have been granted approval for experimental use, there is currently a lack of clinically therapeutic LCN2 molecules. Consequently, there is a need for the identification of additional options and possibilities at any given time. The advent of rapid computer technology has facilitated the extensive utilization of computer-aided drug design (CADD) for the expeditious identification of molecules with favorable target binding potential from a vast array of compounds [24]. This has led to notable cost savings on an array of experimental consumables [25]. In light of the aforementioned factors, the present study was conducted with the primary objective of implementing large-scale virtual screening based on molecular structure.

In this study, we first constructed a receptor–ligand complex-based pharmacophore model and selected RL_5 with Feature Set AHHR and RL_8 with Feature Set AHHH for the virtual screening of 52,765 compounds by analyzing the subject operating characteristic (ROC) curves of the pharmacophore model and comparing the fitness. The molecular docking scores were next screened to obtain the six best-scoring compounds, which helps to quickly screen molecules with potential enzyme binding activity from a large number of compounds, thus reducing the cost of subsequent identification. To obtain the structures of the compounds that interacted better with the LCN2 protein, backbone leaps were performed. The three compounds with the best binding effect were subjected to ADMET (Absorption, Distribution, Metabolism, Excretion, and Toxicity) characterization to predict their drug-forming properties. Following years of development, ADME technology has become a significant approach for the high-throughput virtual prediction and design of new drug candidates. It offers the benefits of greater convenience and reduced cost compared to the traditional method of utilizing animal experiments for ADME property studies. For example, one study used the ADME technique to explore the screening of three HIT compounds that showed better predictions of biological activity and toxicity than positive control compounds [26]. Another study used ADME technique to evaluate toxicological parameters to further screen molecules closer to the drug [18]. In our study, two compounds were identified in ADME characterization as having more desirable drug-forming properties and higher gastrointestinal absorption with some potential for drug formation. Finally, in order to assess the stability of these two candidate molecules in binding to proteins, we performed molecular dynamics calculations, in which the trajectory analysis of the RMSD and RMSF of Compound 69081_50 showed that the compound was able to remain in the protein pocket with minimal fluctuations and stable hydrogen bonding interactions during 100 ns simulations, which indicated that the compound has better stability. This further confirms that Compound 69081_50 has better binding ability to LCN2 protein. Subsequent MM-PBSA calculations also showed that Compound 69081_50 is largely affected by van der Waals forces and its binding free energy results are in good agreement with the expected stability. In addition, docking analyses show that Compound 69081_50 binds other structurally similar members of the lipofuscin family significantly less well than LCN2, making the potential inhibitor Compound 69081_50 specific as an inhibitor of LCN2.

In summary, we successfully pinpointed a promising inhibitor of LCN2 among a vast library of 52,765 diverse and highly bioactive marine natural products, leveraging the power of computer-assisted virtual screening to streamline our search. This will provide a new template for the future optimization of LCN2-associated tumor therapeutic structures.

## 4. Materials and Methods

### 4.1. Protein Preparation

The 3D structure of the LCN2 protein was selected and downloaded from the PDB website (https://www.rcsb.org/, accessed 5 April 2024) at a resolution of 2.20 Å (PDBID: 5NKN). This was then imported into Schrödinger Maestro 11.8 for preliminary protein processing. Specifically, the protein structure was processed for the addition of hydrogen and missing side chains. To refine the protein structure, the PROKA prediction tool was utilized to optimize the protonation state of residues at pH 7.0, enhancing hydrogen bonding capabilities. Additionally, the heavy atoms of the protein were aligned to achieve an RMSD of 0.3 Å, followed by minimization using the OPLS_2005 force field, promoting relaxation throughout the entire structural framework. The protein minimization process, inclusive of all atoms and pure hydrogen, utilizes a conditional termination criterion, dependent on the root-mean-square deviation of heavy atoms from their starting positions. Simultaneously, during the minimization phase, all water molecules are removed while optimizing hydrogen bonding and retaining essential water molecules. Following this, the refined protein molecules are employed for screening LCN2 inhibitors.

### 4.2. Small Molecule Preparation

In this study, the downloaded LCN2 protein (PDBID: 5NKN) was isolated from the small molecule ligands, which were also extracted from the Seaweed Metabolite Database (SWMD) (http://www.swmd.co.in, accessed on 5 April 2024), the Comprehensive Marine Natural Products Database (CMNPD) (https://www.cmnpd.org/, accessed on 5 April 2024), and the Marine Natural Products Database (MNP) (http://docking.umh.es/, accessed on 5 April 2024). A total of 527,650 marine natural compounds were collected and their 3D structures were imported into Schrödinger Maestro 11.8 for further molecular preparation using the Ligprep module. At a pH range of 7.0 ± 2.0, an ionizer was utilized to generate the protonated and ionized configurations of diverse stereoisomers, analogs, and ligands. Subsequently, the energy of the ligands was minimised using the OPLS2005 force field.

To collect the inhibitors of LCN2, we used the large database ChEMBL (https://www.ebi.ac.uk/chembl/g/#search_results/all/query=LCN2, accessed on 10 April 2024) to find them and collected six active small molecules. Due to the small number of active small molecules, we collected 11 more LCN2 inhibitors in the literature [23], which were downloaded from the database PubChem (https://pubchem.ncbi.nlm.nih.gov/, accessed on 10 April 2024) for the next study. The isolation of small molecule ligands from LCN2 protein (PDBID: 5NKN), and the uploading of small molecule ligands to the online website DUD-E (http://dude.docking.org/, accessed on 10 April 2024), were performed, and a total of 51 bait molecules in SMILES format were generated. The format of the small molecules was converted to SDF format by the Openbabel-3.1.1 software for subsequent work.

### 4.3. Pharmacophore Models

#### 4.3.1. Establishment and Validation of Pharmacophore Models

A pharmacophore is a model based on pharmacophore characterization elements. In recent years, the advent of compound databases and the concomitant development of computer technology has led to the widespread adoption of the virtual screening of databases with pharmacophore models. This has become one of the most important means of discovering lead compounds. We imported LCN2 protein (PDBID: 5NKN) into Discovery Studio 2019, set the position where the ligand small molecule is located in the protein as the active center, set the radius to 13, and applied Prepare Protein to preprocess the LCN2 complex. In order to generate a receptor–ligand pharmacophore model, the following settings were applied: Maximum Paracophores = 10, Minimum Features = 4, Maximum Features = 6, and Water Molecules = False. The parallel processing was set to False, while the validation was set to True and the unfolded validation was enabled. To achieve the goal of developing a pharmacophore model rooted in receptor–ligand complexes, we embarked on a process that involved utilizing 17 LCN2 inhibitors as the active ligands and the 51 bait-set molecules as the inactive counterparts. The resulting model was then validated.

#### 4.3.2. Virtual Screening Based on Pharmacophores

**Figure 7:**
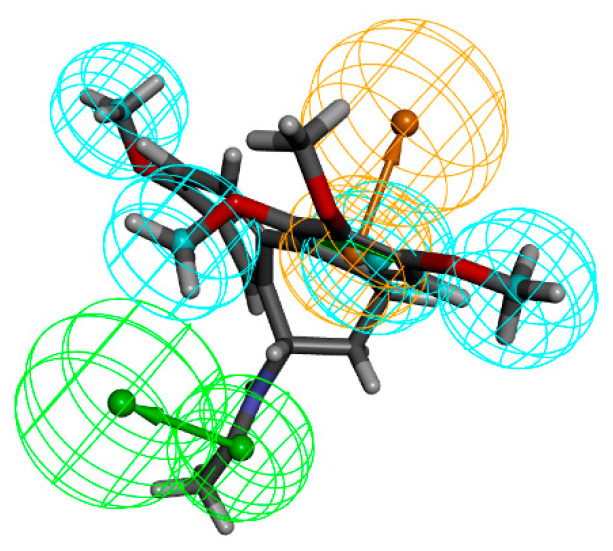
Overlapping effects of 10 receptor–ligand complex-based pharmacophore models.

A total of 10 pharmacophores were generated in the above steps, as shown in Figure 7, and the 2 pharmacophores with better results were selected as RL_5 (with the Feature Set of AHHR) and RL_8 (with the Feature Set of AHHH) to perform the virtual screening based on pharmacophores for 52,765 marine natural compounds. We utilized the Ligand Pharmacophore Mapping module in Discovery Studio 2019, specifically configuring it with the “Best Mapping Only” option activated, ensuring that “Maximum Omitted Features” was zeroed, and selecting both the “Fitting Method” and “Parallel Fitting Method” as “Rigid”, thereby optimizing the process for precision. Furthermore, we opted to disable the “Parallel Processing” feature, maintaining the default settings for all other parameters, in order to commence the virtual screening of marine natural compounds, guided by the pharmacophore model. In the subsequent investigation, a total of 4922 marine natural compounds underwent extensive screening.

### 4.4. Molecular Docking

#### 4.4.1. Molecular Docking Using Maestro

**Figure 8:**
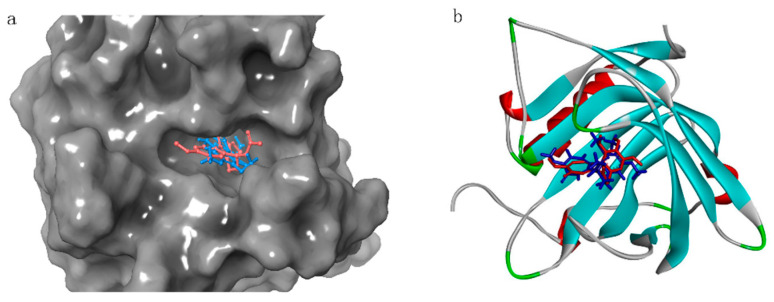
The 5NKN eutectic with redocked ligand in superposition state. (**a**) Maestro: superposition state of 5NKN eutectic with redocked ligand (original eutectic ligand in red, redocked ligand in blue) (RMSD: 1.2575 Å). (**b**) Discovery Studio: superposition state of 5NKN eutectic with redocked ligand (original eutectic ligand in red, redocked ligand in blue) (RMSD: 0.76875 Å).

A collection of studies have demonstrated that molecular docking is an effective method for elucidating the mechanism of target–ligand interaction [27,28,29,30]. To further identify LCN2 inhibitors, ultra-precision docking (XP) was conducted on selected compounds using the LCN2 structure (PDBID: 5NKN) as a reference. To mitigate the potential influence of the nonpolar region within the protein receptor, the receptor’s van der Waals radius scaling factor was calibrated to 1.0, while the partial charge cutoff threshold was adjusted to 0.25. Furthermore, a receptor lattice of dimensions 13 × 13 was constructed with the proto-ligand situated at the centre. For the further evaluation of potential inhibitors of LCN2, the ligand docking tool of the Glide module for Maestro 11.8 was used. However, to ensure the reliability of the structure-based virtual screening process, an additional precision docking (XP) was conducted on the same LCN2 target protein (PDBID: 5NKN) at its eutectic ligand binding site. Before starting the actual molecular docking, this was used to validate the performance of the Glide docking tool. The docked small molecules were superimposed on the original eutectic ligand, and the root-mean-square deviation (RMSD) between them was calculated. The tool was deemed to be a reliable molecular docking tool if the RMSD value was less than 2.0 Å. The RMSD value of the docked small molecules was calculated as the RMSD of the original eutectic ligand. During the validation process, it was found that the re-docked structural attitude is very close to the original eutectic ligand, as shown in Figure 8a. The initial eutectic ligand is marked in red, whereas the repositioned ligand is denoted in blue. The marine natural products were subjected to the next phase of the study upon completion of the validation process. To attenuate the potential energy of the ligand’s nonpolar segment, the small molecules derived from marine natural compounds undergoing screening were configured with a scaling factor of 0.80 and a partial charge cut-off threshold of 0.15. For flexible ligand molecules, the protein engages in further ultra-precise docking (XP) procedures with natural marine compounds. Furthermore, the proto-ligand of LCN2, designated as Compound A, was employed as a positive control small molecule for structure-based virtual screening, thereby facilitating the subsequent phase of virtual screening.

#### 4.4.2. Molecular Docking Using Discovery Studio

Molecular docking represents a firmly established computational approach, extensively utilized in drug discovery endeavors. Docking emerges as a potent instrument, proficient in pinpointing innovative therapeutic compounds, anticipating ligand–target engagements at the molecular level, and elucidating conformation–activity relationships (SARs), all without any prerequisite knowledge of the chemical makeup of other target modulators [31,32]. Moreover, molecular docking serves as a framework for designing pharmaceutical agents, incorporating the receptor’s properties and the receptor–drug interaction patterns. This theoretical simulation approach is extensively utilized to delve into intermolecular interactions and accurately forecast the mode of binding along with the associated affinity. Recently, molecular docking has emerged as a key technology in computer-assisted drug research endeavors. In order to screen efficient LCN2 inhibitors more accurately, we utilized LibDock docking in Discovery Studio 2019 for the next high-throughput screening. Firstly, LCN2 protein (PDBID: 5NKN) was imported into Discovery Studio, the position where the ligand small molecule is located in the protein was set as the active center, and the radius was set to 13. The Dock Ligands (LibDock) module was opened; the Number of Hotspots is set to 100, the Docking Tolerance is set to 0.25, Docking Preferences is set to High Quality, Conformation Method is set to FAST, Minimization Algorithm is set to Do not minimize, Parallel Processing is set to False, and the rest were set to default values. Molecular docking was performed on the ligand small molecules in the proteins and 4922 marine natural compounds screened. Prior to this, to verify the gap between the redocked ligand small molecule and the original ligand conformation, we superimposed and calculated the RMSD of the two small molecules, as shown in Figure 8b. After the docking was completed, the ligand small molecules in the protein were used as a positive control to screen the compounds for the next step.

### 4.5. Fragment Optimization

After analyzing the docking results from both software, six small molecules were selected for fragment optimization. We first selected the replacement fragments for the small molecules and then used the default fragment library of Discovery Studio 2019 (1,495,478 fragments in total) to perform the fragment replacement calculation. Open Replace Fragments in Lead Optimization in Discovery Studio, and, in Fragment Libraries, select One Attachment (Linear), One Attachment (Cyclic), Two Attachments (Linear), Two Attachments (Cyclic), Three Attachments (Linear), Three Attachments (Cyclic), then set Generate Fragment Conformations to False and Minimization to False. Set Generate Fragment Conformations to False, Minimization to False, Parallel Processing to False, expand Advanced, Break Small Fragments to False, Number of Attachments to One, Two, Three, Maximum Number of Atoms to 14, Skip Terminal Bonds False, Prioritize Ligands to True, Maximum Enumerations to 20,000, and Maximum Out of Plane Angle to 10. After setting the above parameters, perform fragment substitution for 6 small molecules. After the substitution was completed, LibDock docking was performed, and the molecules with better results were taken for the next step in the study.

### 4.6. ADME

In addition to the capacity of small molecules to bind to the target, the stability of their metabolic properties in vivo is also a significant indicator of their potential to become a drug. Consequently, it is imperative to ascertain the ADMET characteristics of the compounds in question, with a view to eliminating those with an inadequate capacity to form drugs from the list of alternative compounds. The conventional techniques for evaluating ADME characteristics are conducted through the utilisation of cellular or animal models. However, for the assessment of a substantial number of compounds, these methodologies are not sufficiently efficient or cost-effective [33]. In this study ADME (Absorption, Distribution, Metabolism, and Excretion) screening was performed using the SwissADME online website (http://www.swissadme.ch/, accessed on 10 April 2024) [21], which predicts a range of properties of small molecules by importing smile files of small molecules that include physicochemical parameters, pharmacokinetic profiles, drug similarity, and medicinal chemistry. The accuracy of the predictions ranged from 72% to 94%, meaning that this can help to evaluate the similarity of small molecules and to determine the prospects of small molecules to become orally active drugs in humans.

### 4.7. Molecular Dynamics

We conducted molecular dynamics (MD) simulations to evaluate the stability of the binding between these two molecules and proteins [34,35]. Prior to the simulations, the system was set up using GROMACS 2019.1, developed by Mark Abraham and his team at Uppsala University, Stockholm University, and the Royal Institute of Technology in Sweden [36]. The AMBER99SB-ILDN force field was utilized in the meticulous construction of the protein’s intricate topological framework. The Bio2byte web server was employed for the generation of topology files for molecules (https://www.bio2byte.be/, accessed on 2 July 2024) [37]. The simulation utilized a cubic box with a 1.2 nm radius and the SPC216 water model to apply periodic boundary conditions (PBCs) [38]. We performed 50,000 steps of energy minimization on the system, simulating it at a temperature of 300 Kelvin to optimize its configuration. Following this, the system underwent a rigorous conditioning phase, either maintaining a constant temperature and volume (NVT ensemble) or constant temperature and pressure (NPT ensemble) to ensure stability. Finally, MD simulations with a duration of 100 ns were carried out. Upon the completion of the MD simulation, we analyzed the stability and fluctuations of the system by computing the root-mean-square deviation (RMSD) and the root-mean-square fluctuation of atomic positions (RMSF) from the extracted trajectory coordinates. Furthermore, the gmx-H-bond analysis tool was employed to examine intermolecular hydrogen bonds between LCN2 and ligands [39,40].

### 4.8. MM-PBSA

The MM-PBSA method is a widely employed approach for calculating the free energy of receptor–ligand binding. This technique involves determining the free energy difference between the bound and unbound states of two solvated molecules or comparing free energies among different solvated conformations of a single molecule [41]. The final 10 ns trajectories were extracted from the results of the dynamics simulation for subsequent calculation. The binding free energy is described by the following equation.

G_binding_ = G_complex_ − G_protein_ + G_ligand_

## 5. Conclusions

Two pharmacophore models (RL_5 with Feature Set AHHR and RL_8 with Feature Set AHHH) were developed using receptor–ligand complexes to screen a marine compound database for potential drug candidates for targeting LCN2, a promising therapeutic target for various diseases. A total of 4922 small molecules were selected for molecular docking, with Compound A used as a positive control. After screening, six compounds were subjected to skeleton jumping to increase potency. Three optimized compounds were selected for ADMET property prediction based on molecular docking results. Two compounds underwent 100 ns MD simulations to assess binding stability to LCN2. MM-PBSA calculations on Compound 69081_50 showed good binding free energy results, suggesting it as a potential novel covalent LCN2 inhibitor for targeted therapeutic interventions in LCN2-associated diseases.

## Figures and Tables

**Table 1:** Constructing 10 receptor–ligand complex-based pharmacophore models based on the number of features, feature composition, sensitivity, specificity, ROC curve.

Pharmacophore	Number of Features	Feature Set	Sensitivity	Specificity	Roc Curve
RL_1	5	AHHHR	0.47059	1	0.735
RL_2	5	AHHHH	0.29412	1	0.647
RL_3	4	HHHR	0.47059	1	0.735
RL_4	4	HHHH	0.47059	1	0.735
RL_5	4	AHHR	0.88235	1	0.941
RL_6	4	AHHH	0.82353	1	0.912
RL_7	4	AHHR	0.88235	1	0.941
RL_8	4	AHHH	0.88235	1	0.941
RL_9	4	AHHR	0.88235	1	0.941
RL_10	4	AHHH	0.82353	1	0.912

**Table 2:** Docking scores for six candidate compounds.

Compound	Schrödinger	Discovery Studio
44879	−13.595	151.966
46563	−12.384	150.88
50616	−12.881	158.605
50617	−12.41	156.421
50618	−12.943	156.766
69081	−12.976	146.947
positive control A	−10.994	108.36

**Table 3:** Compound 44879 with Compound 44879_4 structures and Libdock Score.

Compound	Structure	Discovery Studio	Schrödinger
44879	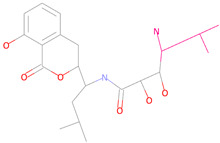	151.966	−13.595
44879_4	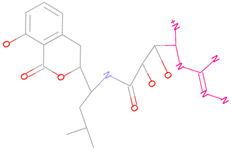	154.759	−15.704

**Table 4:** Compound 69081, Compound 69081_38, Compound 69081_50 structures and Libdock Score.

Compound	Structure	Discovery Studio	Schrödinger
69081	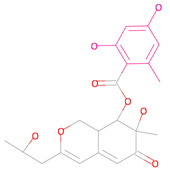	146.947	−12.976
69081_38	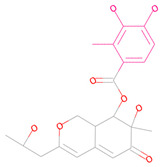	154.690	−13.739
69081_50	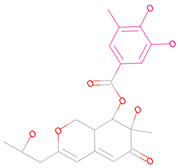	158.584	−13.574

**Table 5:** Three compounds ADME results.

Compound	2D Structure	Molecular Weight (g/mol)	Log Po/w (iLOGP)	BBB Permeant	LogS (ESOL)	Solution	Number of Rotatable Bond	Number of Hydrogen Bond Acceptor	Number of Hydrogen Bond Donor
44879_4	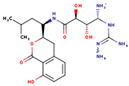	439.49	0.82	NO	−1.92	Low	10	7	8
69081_38	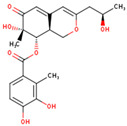	404.41	2.16	NO	−2.83	High	5	8	4
69081_50	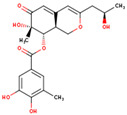	404.41	2.20	NO	−2.83	High	5	8	4

**Table 6:** Binding free energies of two ligands.

Compound	Van Der Waals Energy	Electrostattic Energy	Polar Solvation Energy	SASA Energy	SAV Energy	WCA Energy	Binding Energy
69081_50	−154.108 ± 17.988	−12.725 ± 12.112	76.153 ± 52.103	−12.105 ± 9.125	0.000 ± 0.000	0.000 ± 0.000	−102.785 ± 96.703
A	−159.237 ± 91.219	−23.169 ± 12.274	121.520 ± 56.482	−15.625 ± 8.974	0.000 ± 0.000	0.000 ± 0.000	−76.511 ± 68.704

**Table 7:** LibDock docking scores of the small molecule 69081_50 with lipocalin 2 (LCN2), lipocalin-type PGD2 synthase protein (L-PGDS), human α1-microglobulin (A1M), and retinol-binding protein 4 (RBP4).

Protein	LibDockScore
LCN2	158.5340
L-PGDS	123.9620
A1M	96.1472
RBP4	78.9032

## Data Availability

All data obtained during this study are available from the corresponding author on reasonable request.
